# Haplotype-based interaction of the *PPARGC1A* and *UCP1* genes is associated with impaired fasting glucose or type 2 diabetes mellitus

**DOI:** 10.1097/MD.0000000000006941

**Published:** 2017-06-08

**Authors:** Xiaoting Pei, Li Liu, Jialin Cai, Wenkai Wei, Yan Shen, Yaxuan Wang, Yanzi Chen, Panpan Sun, Mustapha Umar Imam, Zhiguang Ping, Xiaoli Fu

**Affiliations:** a College of Public Health; b School of Basic Medical Sciences, Zhengzhou University, Zhengzhou, China.

**Keywords:** gene, haplotype, impaired fasting glucose, interaction, type 2 diabetes mellitus

## Abstract

The aim of this study is to evaluate the effect of single-nucleotide polymorphisms (SNPs) of the *PPARGC1A* and *UCP1* genes on impaired fasting glucose (IFG) or type 2 diabetes mellitus (T2DM) and the haplotype-based interaction between these genes.

A cross-sectional study was conducted by cluster sampling in Henan province, China. Based on the level of fasting plasma glucose (FPG) and the history of T2DM, the participants were divided into 2 groups; 83 individuals were in the IFG+DM group (those with IFG or T2DM) and 445 individuals were in the NFPG group (those with normal FPG). Kernel canonical correlation analysis (KCCA), a haplotype-based gene-gene interaction method, which can increase the biological interpretability and extract nonlinear characteristics of SNPs, was used to analyze the correlation and interaction between *PPARGC1A* and *UCP1* genes.

The age, BMI, total cholesterol and triglycerides were statistically different between 2 groups (*P* ≤ .001). Haplotype analysis showed no significant difference in frequency distribution between the 2 groups when the *PPARGC1A* or *UCP1* gene was tested (*P* > .05). KCCA analysis showed that the maximum kernel canonical correlation coefficient of the *PPARGC1A* and *UCP1* genes was 0.9977 and 0.9995 in the IFG+DM and NPFG groups, respectively. A haplotype-based gene–gene interaction was observed significantly (*U* = −6.28, *P* < .001), indicating the possibility of an interaction between haplotype AAG of the *PPARGC1A* gene and haplotypes CTCG (odds ratio [OR] = 1.745, 95% confidence interval [95% CI] 1.069–2.847) and CTCA (OR = 0.239, 95% CI 0.060–0.958) of the *UCP1* gene.

Haplotype-based interaction between the *PPARGC1A* and *UCP1* genes is associated with IFG or T2DM among residents in Henan, China.

## 1 Introduction

Impaired fasting glucose (IFG) is indicative of an abnormal health status, but it does not meet the diagnostic criteria for diabetes mellitus (DM).^[1]^ IFG is a risk factor for cardiovascular disease,^[2]^ and individuals with IFG have 4 to 6 times higher risk of developing DM than healthy individuals.^[3]^ Patients in whom diabetes is not efficiently controlled have a significantly higher risk of developing complications such as diabetic foot, diabetic nephropathy, and diabetic retinopathy, which seriously affect the patient's quality of life and life expectancy.^[4–6]^ Data from the International Diabetes Federation(IDF) showed that in 2015 there were about 415 million people with diabetes, and this is expected to rise to 642 million by 2040,^[7,8]^ which will make DM the third leading noncommunicable chronic disease after cerebrovascular diseases and cancers. In China, the prevalence of DM is 9.7%, and that of IFG is even higher at 15.5%.^[9,10]^ Fortunately, unlike irreversible DM, patients with IFG can be treated with the appropriate interventions, thereby reducing the incidence of DM and greatly improving the living quality of individuals.

The peroxisome proliferator activated receptor gamma coactivator-1 alpha (PPARGC1A) is a nuclear transcriptional factors, which plays a key role in metabolism of carbohydrates and lipids.^[11]^ The single-nucleotide polymorphisms (SNPs) of the human *PPARGC1A* gene are associated with the development of type 2 diabetes mellitus (T2DM).^[12–14]^ The uncoupling protein 1 (UCP1) is predominantly expressed in brown adipose tissue, where it mediates nonshivering thermogenesis and energy thermodynamics, and thus plays an important role in resisting the development of obesity in humans.^[15]^ Evidence shows that SNPs of the *UCP1*gene are associated with the development of obesity and T2DM.^[16]^ Thus, there might be an interaction between the *PPARGC1A* and *UCP1* genes, because both genes can regulate the metabolism of lipids and can impact on the development of T2DM. There have been reports of the relationship between the *PPARGC1A* or *UCP1* gene and T2DM,^[14,16]^ but neither the relationship between these 2 genes and IFG nor the correlation and interaction between the *PPARGC1A* and *UCP1*genes have been reported. The interaction of genes was performed by multifactor dimensionality reduction (MDR), logistic regression model, and crossover analysis in traditional studies, which are based on SNP–SNP interaction, so the results may be affected by the degree of linkage disequilibrium within SNP. In this study, we used the kernel canonical correlation analysis (KCCA) method to determine the interaction of *PPARGC1A* and *UCP1* genes. KCCA, introduced by Akaho in 2001, is a method that is based on haplotypes and analyzes interaction between 2 whole genes. KCCA can increase the biological interpretability due to dimensional reduction. Moreover, KCCA can extract nonlinear characteristics of SNPs between 2 whole genes and conduct interaction analysis more accurately. It no longer requires the normal distributional assumption on data.^[17,18]^ Therefore, we used data from a cross-sectional study in Henan province to establish the haplotypes of the *PPARGC1A* and *UCP1* genes and then analyze the correlation and interaction between these genes by the KCCA method to explore their relationship with IFG or T2DM.

## 2 Materials and methods

### 2.1 Study participants

A 2-stage cluster sampling investigation was conducted in Xin’an, a county of Luoyang City in Henan province, China in 2011. In the first stage, 5 out of 11 towns were selected randomly, while in the second stage, 1 village was selected from each town by cluster sampling. The individuals who voluntarily participated in the study and met the inclusion criteria filled out a questionnaire and had physical examinations and laboratory tests. The participants were divided into the IFG+DM group (whose FPG ≥6.1 mmol/L and T2DM patients taking or not taking hypoglycemic agents) and the normal FPG group (NFPG, whose FPG < 6.1 mmol/L, excluding those taking hypoglycemic agents even if their FPG was below 6.1 mmol/L).^[19]^ The study was approved by the Ethics Committee of Zhengzhou University and informed consented was sought from all participants.

### 2.2 Data collection

A questionnaire was completed by each participant to obtain information of demographic characteristics and lifestyle risk factors. Height and weight of each participant were measured according to the unified standards by trained staff, and then body mass index (BMI = weight/height^2^ (kg/m^2^)) was calculated. Current smoking was defined as having smoked 100 cigarettes and smoking cigarettes currently. Current drinking was defined as alcohol intake more than once per month during the past 12 months. The cubital venous blood was collected and stored at −20°C to detect the levels of FPG, serum total cholesterol (TC), triglyceride (TG), low-density lipoprotein (LDL), and high-density lipoprotein (HDL) by trained laboratory technicians. DNA was extracted from the peripheral blood for the analysis of SNPs of the *PPARGC1A* and *UCP1* genes.

### 2.3 Selection of SNPs

Three SNPs of the *PPARGC1A* gene and 4 SNPs of the *UCP1* gene were selected (Table 1). The selection criteria were that the SNPs fulfilled 1 of the following conditions^[20]^: the gene segment of SNPs can cause functional changes; SNPs with established association with IFG or T2DM; and it is heterozygous and its minor allele frequency (MAF) is greater than 5%.

**Table 1:**
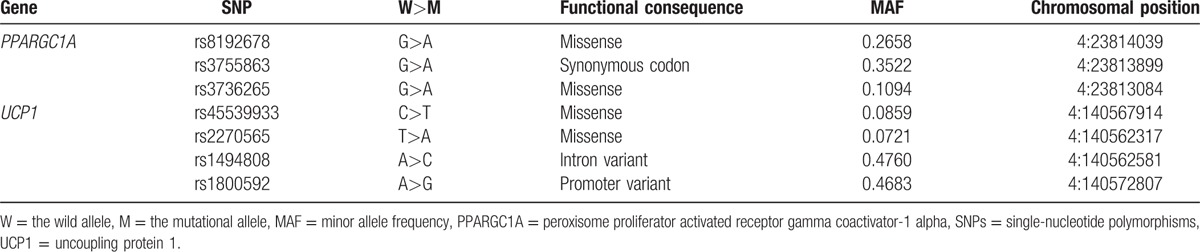
Information on the *PPARGC1A* and *UCP1* SNPs.

### 2.4 Genotyping of the *PPARGC1A* and *UCP1* genes

DNA was extracted using the genomic DNA purification kit (Promega, Madison, WI) according to manufacturer's instruction and the content of DNA was detected by gel electrophoresis. Ligase detection reaction (LDR) was used to determine the genotype of each SNP. Primer information is shown in Table 2

**Table 2:**
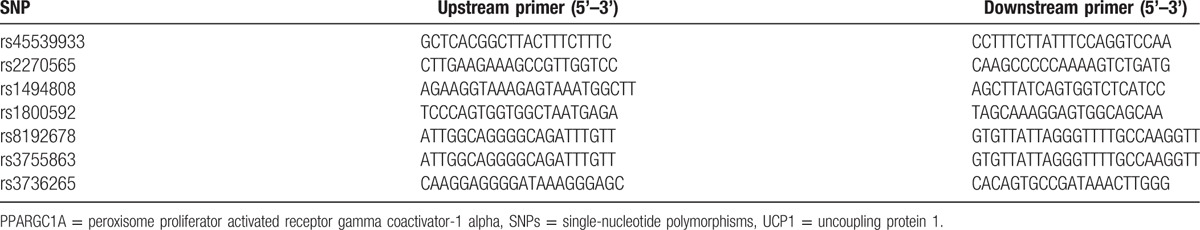
Primers used for detection of the *PPARGC1A* and *UCP1* SNPs.

### 2.5 Statistical analyses

Statistical analyses were conducted using the IBM SPSS 21.0 (SPSS Inc, Chicago, IL) and R software 3.3.1. Demographic and biochemical characteristics of participants were described in means and standard deviation (
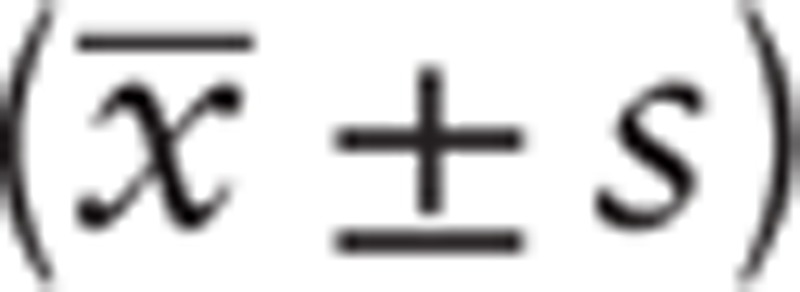
) for quantitative variables and frequencies for qualitative variables. The χ^2^ test was performed to determine differences in sex, smoking, and drinking status between 2 groups. Unpaired *t*-test or Wilcoxon rank sum test (data that were not normally distributed by the Shapiro–Wilk test) was used to compare the differences of age, BMI, and biochemical characteristics between the 2 groups. The SNPassoc package^[21]^ of R software was used to analyze the associations between genes and IFG or T2DM. The linkage disequilibrium (LD) analysis and the haplotype analysis were carried out by SHEsis online (http://analysis.bio-x.cn/myAnalysis.php).^[20]^ Kernlab package ^[22]^ of R software was used for KCCA. Deviations from Hardy–Weinberg equilibrium (HWE) were assessed by the *χ*^*2*^ tests. All *P*-values were 2-tailed, and the level of significance was set at *α* = 0.05.

## 3 Results

### 3.1 The characteristics of participants

A total of 583 people were investigated. Excluding invalid questionnaires and people with genetic relationship within 3 generations according to the research objectives and methods, we identified 528 participants, including 198 males and 330 females. There were 445 in the NFPG group and 83 in the IFG+DM group. The results showed that there were no statistical differences between the 2 groups in sex, smoking, drinking status, and the levels of HDL and LDL (*P* > .05). On the other hand, the age, BMI, and the levels of TC, TG, and FPG were statistically different between the 2 groups (*P *≤* *.001), with those of the IFG+DM group being higher than the NFPG group (Table 3).

**Table 3:**
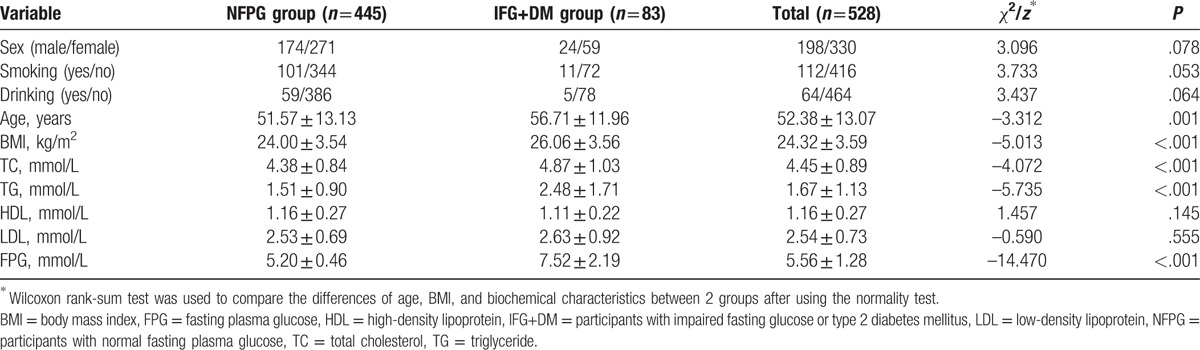
Comparison of demographic and biochemical characteristics between the 2 groups.

### 3.2 Distribution of genotypes and alleles and the test of Hardy–Weinberg equilibrium

Seven SNPs were genotyped in all the 528 participants. All of the SNPs showed no statistical deviation from the test of HWE (*P* > .05) (Table 4), which indicated that the population of this study was representative.

**Table 4:**
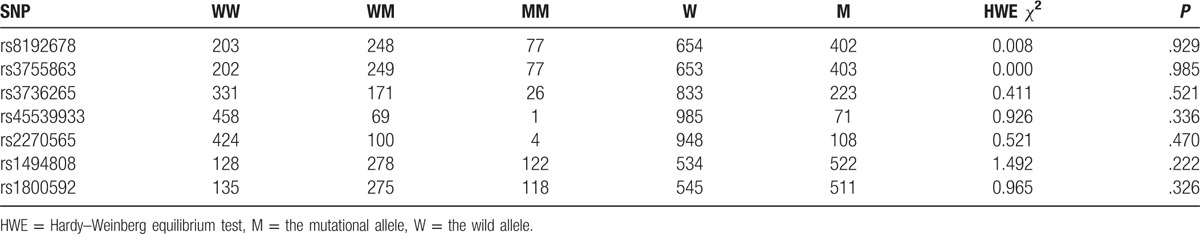
Distribution of genotypes and alleles and the test of Hardy–Weinberg equilibrium.

### 3.3 Associations between the *PPARGC1A* and *UCP1* gene polymorphisms and IFG or T2DM

When considering the genotype distribution of the 7 SNPs, no significant differences were observed between the IFG+DM and NFPG groups, using the codominant, dominant, and recessive genetic models, even after adjusting for age, sex, drinking status, and BMI (*P* > .05). There were also no significant differences in allele frequencies (*P* > .05) (Table 5).

**Table 5:**
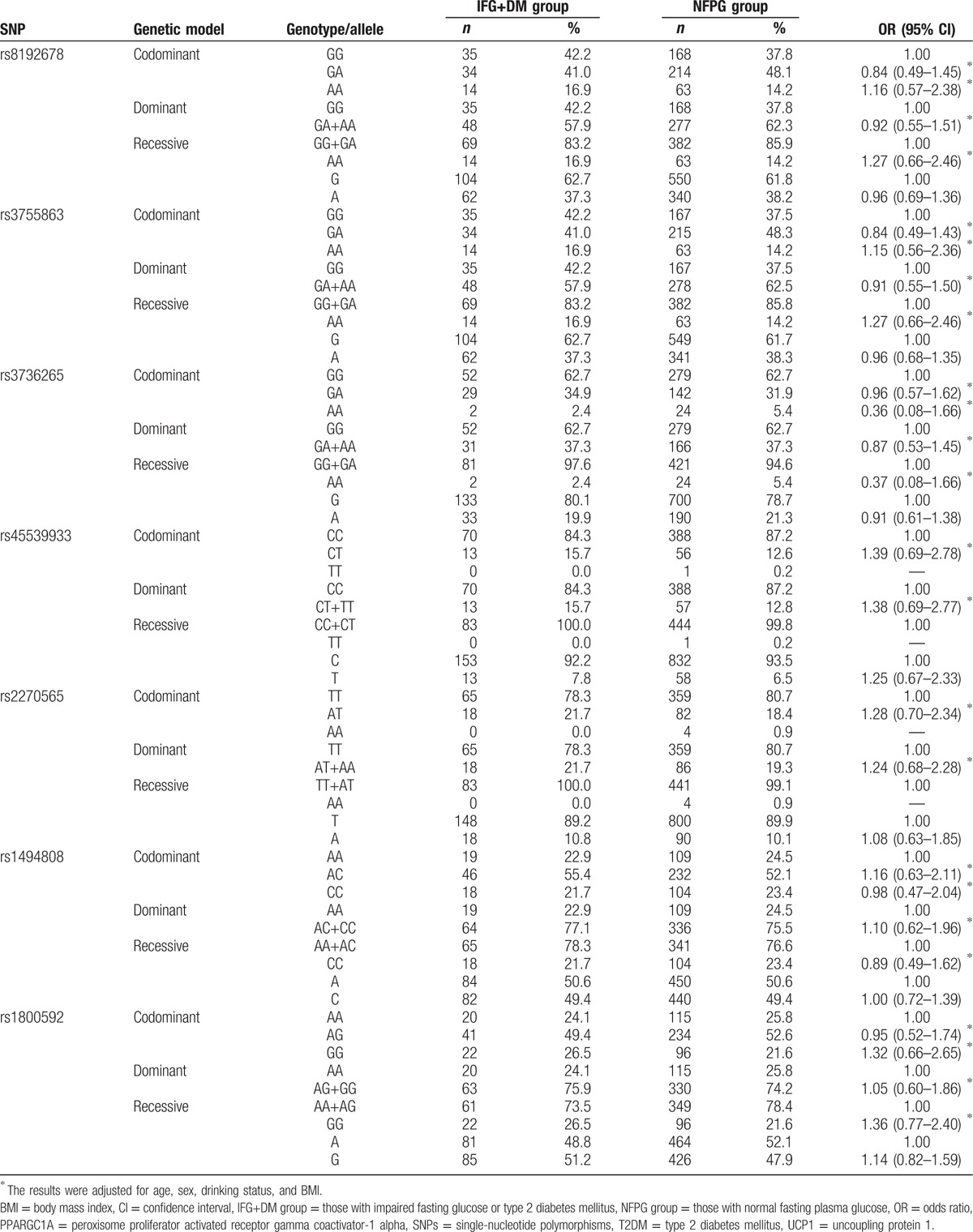
Associations of genotypes or allele frequencies of the *PPARGC1A* and *UCP1* SNPs with IFG or T2DM.

### 3.4 Haplotype analyses

**Figure 1:**
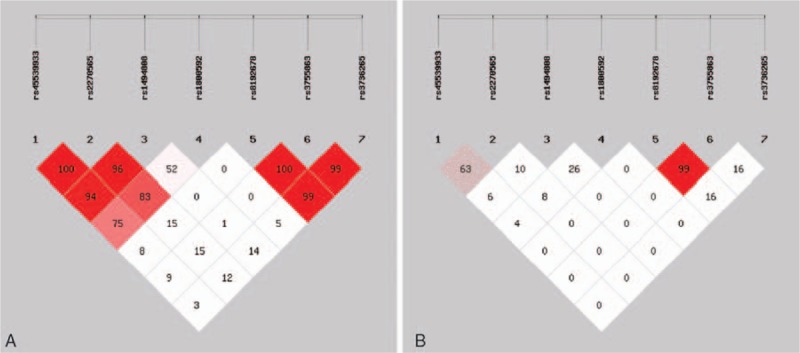
Analysis of linkage disequilibrium of the 7 SNPs. (A) The color and figure were determined by the value of *D*′. (B) The color and figure were determined by the value of *r*^2^. SNPs = single-nucleotide polymorphisms.

The human *PPARGC1A* and *UCP1* genes are both located on chromosome 4. The SHEsis online was used to analyze the degree of linkage disequilibrium of the 7 SNPs and haplotypes in this study. It was observed that there was linkage disequilibrium among 3 SNPs of the *PPARGC1A* gene and 4 SNPs of the *UCP1* gene. However, there was linkage equilibrium between the *PPARGC1A* and *UCP1* genes (Fig. 1). For SNPs of the *PPARGC1A* gene, 3 haplotypes each with a frequency greater than 1% were detected due to linkage disequilibrium, and 6 haplotypes for 4 SNPs of the *UCP1* gene were also detected. The 3 linked haplotypes of the *PPARGC1A* gene from left to right were the alleles of rs8192678, rs3755863, and rs3736265, respectively, while the 4 linked haplotypes of the *UCP1* gene from left to right were the alleles of rs45539933, rs2270565, rs1494808, and rs1800592, respectively. Haplotype analysis showed that there were no significant differences in frequency distribution between the 2 groups for both the *PPARGC1A* and *UCP1* genes (*P* > .05) (Table 6).

**Table 6:**
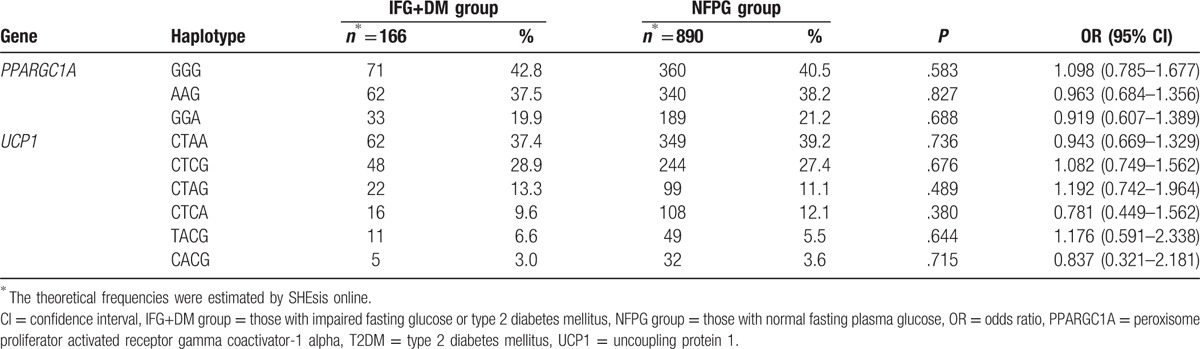
Associations between the *PPARGC1A* and *UCP1* gene haplotypes and IFG or T2DM.

### 3.5 Correlation and interaction between the *PPARGC1A* and *UCP1* genes

The haplotype-based gene–gene interaction method, KCCA was used to analyze the correlation and interaction between the *PPARGC1A* and *UCP1* SNPs. In the IFG+DM and NPFG groups, the maximum kernel canonical correlation coefficients (KCCC) of the *PPARGC1A* and *UCP1* genes were 0.9977 (*r*_*D*_ = 0.9977) and 0.9995 (*r*_*C*_ = 0.9995), respectively. Based on the difference of KCCC of the 2 genes between the 2 groups, a Fisher *r-to-z* transformation was proposed for testing the interaction between the 2 genes on disease outcomes. This transformation was done to *r*_*D*_ and *r*_*C*_, that is, Z_*D*_ = ½(ln(1 + *r*_*D*_) - ln(1 - *r*_*D*_)), Z_*C*_ = ½(ln(1 + *r*_*C*_) - ln(1 - *r*_*C*_)). ^[23]^ The KCCU statistic ^[24]^ was taken as a measure of haplotype-based gene–gene interaction in the IFG+DM and NFPG groups, which can be defined as 
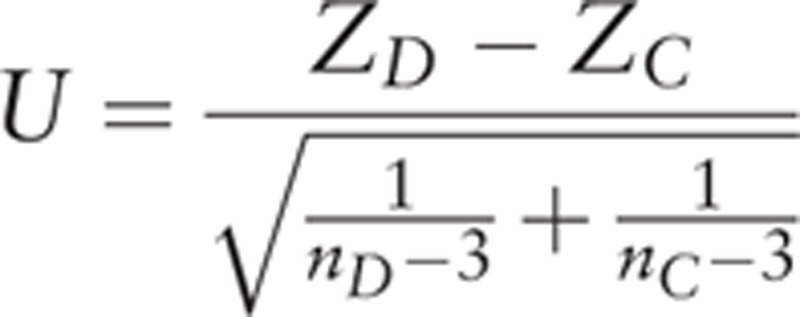


The result of this study was *U* = −6.28, *P* < .001, suggesting that the interaction of the *PPARGC1A* and *UCP1* genes affects IFG or T2DM. To examine whether this interaction does exist, we constructed haplotypes using SHEsis online. The haplotypes of these 2 genes from left to right were the alleles of rs8192678, rs3755863, rs3736265, rs45539933, rs2270565, rs1494808, and rs1800592, respectively. Thirteen haplotypes, each with a frequency greater than 1%, were detected. The results of the haplotype analysis showed that there was an interaction between haplotype AAG of the *PPARGC1A* gene and haplotypes CTCG (*P* = .024, odds ratio [OR] = 1.745, 95% confidence interval [95% CI] 1.069–2.847) and CTCA (*P* = .028, OR = 0.239, 95% CI 0.060–0.958) of the *UCP1* gene (Table 7). This suggested that the individuals with the haplotypes AAG (*PPARGC1A* gene) and CTCG (*UCP1* gene) have higher susceptibility to IFG or T2DM, while those with haplotypes of AAG (*PPARGC1A* gene) and CTCA (*UCP1* gene) have lower susceptibility to IFG or T2DM. In other words, the interaction between the *PPARGC1A* and *UCP1* genes is associated with IFG or T2DM in Chinese Han population.

**Table 7:**
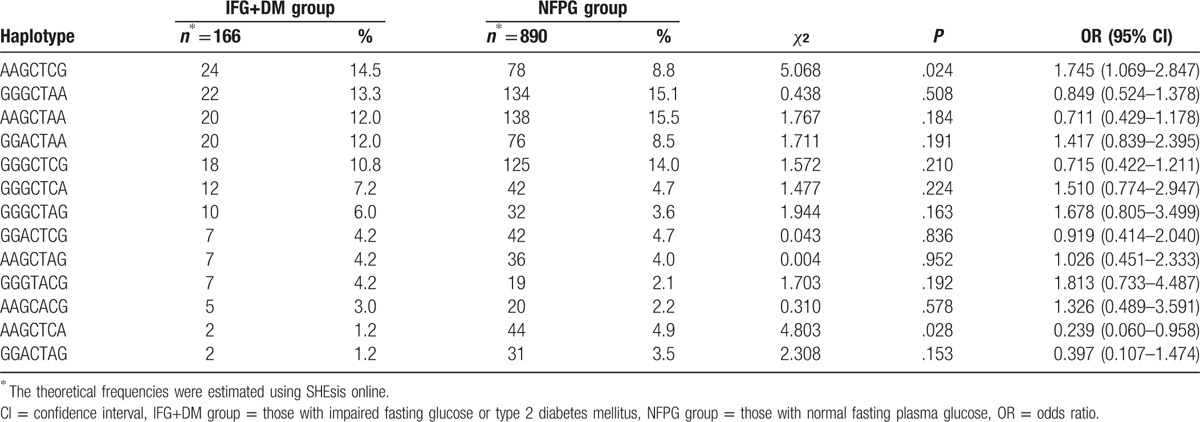
Distribution of haplotypes of the *PPARGC1A* and *UCP1* genes in the IFG+DM and NFPG groups.

## 4 Discussion

There is a better understanding of the pathogenesis of T2DM, due to which more efficient control measures are being adopted. However, environmental and lifestyle factors have largely kept the prevalence of T2DM high and the age of onset is becoming younger than before.^[7]^ PPARGC1A is a transcriptional factor that belongs to the PPAR family and is involved in regulating energy metabolism, fatty acid oxidation, and glucose utilization.^[25–27]^ On the other hand, UCP1 exerts influence on obesity, T2DM, and other noncommunicable chronic diseases by regulating the metabolism of carbohydrates and lipids.^[16]^ In this study, we selected the loci of rs3755863, rs3736265, rs45539933, rs2270565, and rs1494808 from the *PPARGC1A* and *UCP1* genes and evaluated the relationship between these loci and IFG or T2DM as well as the interaction between the *PPARGC1A* and *UCP1* genes, which have never been reported either for the Chinese or the global population. In addition, we considered both FPG and T2DM as groups, which is different from previous studies. It is helpful to detect and diagnose the early stages of T2DM, so that appropriate measures can be instituted to reverse the disease or slow down its progression, thereby reducing the prevalence of T2DM.

This study indicated that the age, BMI, and the levels of TC, TG, and FPG of those with IFG and/or T2DM were significantly higher than those without any of these conditions. These findings are similar to those from a study in Sichuan, China by Song et al.^[28]^ Additionally, the present study detected no difference in the genotypes and allele distribution of the 3 SNPs of the *PPARGC1A* gene (rs8192678, rs3755863, and rs3736265) between the IFG+DM and NFPG groups even after adjusting for age, sex, BMI, and drinking status, suggesting that the polymorphisms of the *PPARGC1A* gene were not associated with IFG or T2DM. A meta-analysis by Yang et al^[29]^ indicated that the polymorphisms of Gly482Ser (rs8192678) and Thr394Thr (rs2970847) in the *PPARGC1A* gene were significantly associated with the risk of T2DM, especially in the Indian population, with OR values (95% CI) of 1.19(1.05–1.34) and 1.33(1.34–1.70), respectively. No relationship was observed between the Thr612Met locus and the risk of T2DM. Zhang et al^[30]^ found that the polymorphisms of Thr394Thr (rs2970847) and Gly482Ser (rs8192678) in the *PPARGC1A* gene were associated with the therapeutic efficacy of multiple-dose rosiglitazone in Chinese patients with T2DM. Another study suggested that the C allele of rs2946386 in the promoter region of the *PPARGC1A* gene was not associated with T2DM.^[14]^ In the present study, susceptibility analysis on the *UCP1* gene revealed that the genotypes and allele distribution of the 4 SNPs in the *UCP1* gene (rs45539933, rs2270565, rs1494808 and rs1800592) were also not significantly different between the IFG+DM and NFPG groups, suggesting no association between these polymorphisms and IFG or T2DM. Nicoletti et al^[16]^ analyzed the polymorphic locus of -3826 A>G (rs1800592) in the *UCP1* gene and found that it increased the risk of T2DM in obese individuals. Besides that, a study by Zhang et al^[31]^ showed that the allele G and genotype GG of the SNP rs1800592 in the *UCP1* gene was associated with the increased risk of proliferative diabetic retinopathy (PDR) in the Chinese population with T2DM. Reasons for the inconsistent results may include: the occurrence and development of diseases are not only associated with the gene but may be modified by lifestyle and environment such as differences in nutritional and economic status.^[32]^ All of these may have an impact on the level of blood glucose, thus leading to the different results. We considered both the level of FPG and the history of T2DM to divide participants into 2 groups in this study, while in previous studies, most of the researchers grouped participants according to the diagnostic criteria of T2DM or gestational diabetes mellitus. Different stages or types of DM may have different impacts on the body, which may lead to inconsistent results in previous studies. Each study selected a variety of SNPs, and different SNPs even if they are in the same gene may have a different degree of linkage disequilibrium and interaction, so that the effect of genes on disease is diverse. The various sample sizes and composition of each group may have introduced different sampling errors and confounding factors, which may affect the results of the studies.

The occurrence and development of disease usually depend on the interrelation of several SNPs or genes not only a single gene locus. Genetic susceptibility is often inherited in the form of haplotypes, and gene–gene interaction may have diverse effects on extrinsic phenotype.^[33]^ Comparing with the traditional methods, KCCA is based on haplotypes and analyzes interaction between 2 whole genes, so it may better capture the true underlying genotypic–proteinic–phenotypic relationship by dimensional reduction under multi-locus SNP models. Moreover, KCCA can extract nonlinear characteristics of SNPs between 2 whole genes and conduct interaction analysis more accurately and reliably.^[17,34]^ In addition, the relationship between the *PPARGC1A* or *UCP1* gene and IFG or T2DM, and the interaction between these genes toward the development of these disease conditions have not been documented elsewhere.

In the present study, the distribution of haplotypes in the IFG+DM and NFPG groups was analyzed by constructing haplotypes of the *PPARGC1A* and *UCP1* genes, and no significant differences were observed between the 2 groups for either the *PPARGC1A* or *UCP1* gene. It suggested that there was no association between haplotypes of both of the genes and IFG or T2DM among residents of Luoyang, Henan province. The interaction of the *PPARGC1A* and *UCP1* genes on IFG or T2DM was analyzed by calculating the KCCC between the *PPARGC1A* and *UCP1* genes. The KCCA showed that the *PPARGC1A* gene highly correlated with the *UCP1* gene in both IFG+DM and NFPG groups, and an interaction between these genes affected the development of IFG or T2DM. Moreover, this result was also confirmed by the analysis of “haplotypes” composed of the *PPARGC1A* and *UCP1* genes. Furthermore, haplotype analysis showed that there was an interaction between haplotype AAG of the *PPARGC1A* gene and haplotypes CTCG and CTCA of the *UCP1* gene. The interaction of the haplotypes AAG (*PPARGC1A* gene) and CTCG (*UCP1* gene) increased the susceptibility of IFG or T2DM, while that of AAG (*PPARGC1A* gene) and CTCA (*UCP1* gene) lowered risk of IFG or T2DM. A study on backcross progenies of mice reported that there was a strong correlation between the levels of the PPARGC1A and UCP1 mRNA, with a correlation coefficient of 0.85.^[35]^ Another study showed that *PPARGC1A* can regulate the expression of UCP1.^[36]^

The main limitations of this study are the limited sample size especially in the IFG+DM group and that the study participants were drawn from 1 province. It would be helpful to examine these findings in larger samples in future studies. Such larger studies are important in order to establish the definitive association between the *UCPI* and *PPARGC1A* genes and IFG or T2DM, for a clearer picture on their public health significance.

In conclusion, the haplotype-based interaction between the *PPARGC1A* and *UCP1* genes is associated with IFG or T2DM among the residents of Henan province, China. Individuals with the haplotype AAG (*PPARGC1A* gene) and CTCG (*UCP1* gene) have increased susceptibility to IFG or T2DM, while those with haplotype AAG (*PPARGC1A* gene) and CTCA (*UCP1* gene) have a lower risk of IFG or T2DM.

## Acknowledgments

This work was supported by the National Natural Science Foundation of China (grant numbers 81001280, 81202277, 81373096); the Key Research Project for Colleges and Universities in Henan Province (grant numbers 16A330003). The authors thank the volunteers in this project for their devotion to the study of impaired fasting glucose or type 2 diabetes mellitus.
